# Intrapartum sonographic evaluation of fetal head descent in relation to maternal position: comparison between dorsal lithotomy and kneeling squat positions

**DOI:** 10.1002/uog.70230

**Published:** 2026-04-15

**Authors:** C. Melito, L. Rizzo, L. Yurhel, E. Corno, S. Renzetti, M. C. Malvezzi, F. Frati, S. Neri, T. Ghi, A. Dall'Asta

**Affiliations:** ^1^ Department of Medicine and Surgery, Obstetrics and Gynecology Unit University of Parma Parma Italy; ^2^ Department of Medicine and Surgery, Unit of Biostatistics and Epidemiology University of Parma Parma Italy; ^3^ Department of Life Sciences and Public Health Catholic University of Sacred Heart Rome Italy; ^4^ Department of Woman and Child Health and Public Health Fondazione Policlinico Universitario Agostino Gemelli IRCCS Rome Italy

**Keywords:** dorsal lithotomy position, fetal head station, kneeling squat position, latent phase of labor, occiput position, transperineal ultrasound

## Abstract

**Objective:**

To evaluate the impact of maternal position on the sonographic indicators of fetal head descent in the latent phase of the second stage of labor.

**Methods:**

This was a prospective, single‐center, cohort study conducted at the University of Parma, Parma, Italy, between November 2023 and October 2024, including a consecutive series of non‐anomalous, low‐risk, singleton pregnancies at ≥ 37 + 0 weeks' gestation in the latent phase of the second stage of labor. Sonographic assessment of the fetal head position and station was performed by two dedicated and trained research midwives; the former was evaluated using transabdominal ultrasound, and the latter by measuring the head‐to‐perineum distance (HPD) and the angle of progression (AoP) on transperineal ultrasound. The sonographic indicators of fetal head station were measured between uterine contractions, with the mother first in the dorsal lithotomy position and then in the kneeling squat position. The Wilcoxon signed‐rank test and linear mixed‐effects models were applied to compare the measurements of AoP and HPD between the dorsal lithotomy and kneeling squat positions. Generalized additive models (GAMs) were utilized to describe the relationship and estimate the predicted change in AoP and HPD when transitioning from the dorsal lithotomy to the kneeling squat position.

**Results:**

Overall, 55 patients underwent measurement of the sonographic indicators of fetal head station in both maternal positions. Occiput posterior position was recorded in only two cases (3.6%), with the remaining cases in occiput anterior position. Both the Wilcoxon signed‐ranked test and linear mixed‐effects models showed that HPD was significantly shorter when measured in the kneeling squat compared with the dorsal lithotomy position (30.3 ± 8.2 *vs* 35.5 ± 8.9 mm, *P* < 0.01). Consistently, a wider mean AoP was recorded in the kneeling squat compared with the dorsal lithotomy position (135.4° ± 15.1° *vs* 125.6° ± 11.9°, *P* < 0.01). GAMs highlighted an increasing non‐linear relationship between measurements of AoP and HPD obtained in the dorsal lithotomy and kneeling squat positions.

**Conclusion:**

During the latent phase of the second stage of labor, maternal mobilization into upright positions, such as the kneeling squat position, is associated with more favorable sonographic indicators of fetal head station. However, this study supports such findings only in the event of fetal occiput anterior position. Sonographic studies with larger cohorts are warranted to evaluate the clinical relevance of these findings, as well as the role of maternal mobilization in the second stage of labor in relation to the position of the fetal occiput. © 2026 The Author(s). *Ultrasound in Obstetrics & Gynecology* published by John Wiley & Sons Ltd on behalf of International Society of Ultrasound in Obstetrics and Gynecology.

## INTRODUCTION

Fetal head descent is a critical component of the birthing process and is commonly assessed by digital vaginal examination to determine the fetal head station in relation to the ischial spines^1^. However, such evaluation has been shown to be highly inaccurate and poorly reproducible^1^, ^2^, ^3^. Intrapartum ultrasound has been proposed as a reliable and highly reproducible alternative for assessing fetal head station^4^, ^5^. Additionally, the use of ultrasound during labor is associated with a low risk of infection^6^, ^7^, is well tolerated by patients^8^ and can predict the success of an instrumental delivery^9^, ^10^, ^11^, ^12^ and labor outcome in the event of labor dystocia during the first or second stage of labor^13^, ^14^, ^15^.

Birthing positions include upright (vertical) and neutral (horizontal) positions and are classified depending on the angle formed between the horizontal plane and the line connecting the midpoints of the third and fifth lumbar vertebrae^16^, ^17^, ^18^, ^19^. The former includes squatting/kneeling, squatting, seated, suspended and standing positions, while the latter includes dorsal lithotomy, gynecological, supine and lateral positions^16^, ^20^. The change of maternal position during labor is proposed as a non‐medical strategy to help facilitate the progression of labor and childbirth^21^, ^22^, ^23^. Although evidence from high‐quality randomized controlled trials (RCTs) is lacking, the World Health Organization endorses a maternal upright position during labor^24^, given its association with a reduction in the rates of episiotomy, postpartum hemorrhage, fetal heart rate abnormalities and obstetric intervention for labor dystocia^16^, as well as the documented shorter durations of the first and second stages of labor in a squatting position compared with the dorsal lithotomy position^25^, ^26^. Upright positions have also been proposed to reduce the incidence of major perineal tears^27^. However, there is controversy on this topic as other studies have reported a lower rate of obstetric anal sphincter injury in relation to the improved perineal support in the dorsal lithotomy position^16^.

The rationale behind adopting upright (vertical) positions during labor is that, from a biomechanical perspective, they increase the size of the pelvis, especially the bispinous diameter^28^, ^29^, and, together with gravity, contribute to the force facilitating fetal head descent^30^. Moreover, in these positions, the sacrum and coccyx are free to move, in contrast to the supine position in which the force of the bed beneath the sacrum closes the pelvis^31^. In such context, a previous study evaluating the sonographic indicators of fetal head station in the lateral *vs* the semirecumbent maternal position^32^ reported small but significant differences in fetal head station in relation to the maternal position. The aim of this study was to evaluate and compare the sonographic indicators of fetal head station in the second stage of labor in the dorsal lithotomy *vs* the kneeling squat position.

## METHODS

**Figure 1:**
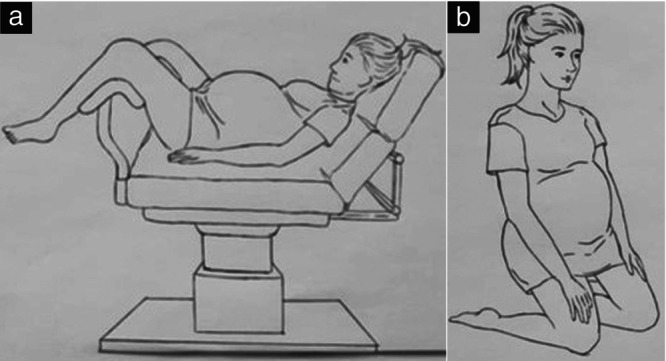
Illustration of (a) dorsal lithotomy and (b) kneeling squat maternal positions.

This was a prospective, single‐center, observational, cohort study conducted at the University Hospital of Parma, Parma, Italy, between 1 November 2023 and 31 October 2024. A consecutive series of non‐anomalous, low‐risk, singleton pregnancies at ≥ 37 + 0 weeks' gestation in the latent phase of the second stage of labor following normal labor progression were included. The eligible cases underwent sonographic assessment of fetal head station via transperineal ultrasound examination, first in the dorsal lithotomy position and then, sequentially, in the kneeling squat position (Figure 1). Ultrasound examination was performed by two research midwives (L.R. and L.Y.) who had previously completed dedicated training in intrapartum ultrasound. Following sonographic confirmation of an empty maternal bladder, sonographic evaluation of fetal head descent was performed by measuring transperineally the angle of progression (AoP) and the head‐to‐perineum distance (HPD). The transducer was first positioned longitudinally between the labia majora to assess the AoP, which was measured as the angle between a line at the long axis of the pubic symphysis and a second line extending tangentially from the most inferior portion of the pubic symphysis to the fetal skull contour^33^. Subsequently, the HPD was measured in the axial plane as the shortest distance between the outer bony limit of the fetal skull and the transducer edge^33^. Measurements were performed between uterine contractions, first in the dorsal lithotomy position and then, following maternal mobilization, in the kneeling squat position. The sequential measurements of the sonographic indicators of fetal head station in the two positions were obtained within the shortest feasible timeframe (within 5 min in all cases). At the time of enrolment, the position of the fetal head was assessed using transabdominal ultrasound by placing the probe transversely over the maternal suprapubic region and categorized as described previously^33^. Written informed consent for study inclusion was obtained prior to enrolment.

Labor characteristics and maternal demographics were retrieved from patient medical records and included maternal age, ethnicity, parity, pregestational body mass index, gestational age at presentation, induction of labor and analgesia, in addition to labor outcomes including labor duration, mode of delivery and postpartum hemorrhage. The primary outcome of the study was to investigate whether fetal head descent, as evaluated by intrapartum transperineal ultrasound, differs according to maternal position during labor, more specifically between the dorsal lithotomy and the kneeling squat positions.

### Statistical analysis

For descriptive analysis, normally distributed continuous variables are presented as mean ± SD while non‐normally distributed continuous variables are reported as median and range. Categorical variables are expressed as *n* (%). The Wilcoxon signed‐rank test for paired data was used to assess the differences in measurements of AoP and HPD between the dorsal lithotomy and the kneeling squat positions. Linear mixed‐effects models were applied to assess the differences in measurements of AoP and HPD between the two positions, adjusting for ethnicity, fetal occiput position and parity. The regression parameter (β) associated with maternal position represented the expected change in the AoP and HPD when switching from the reference position (dorsal lithotomy) to the comparison position (kneeling squat), holding all covariates constant and accounting for repeated measures through the random effects. Generalized additive models (GAMs) were applied to describe the relationship between the measurements obtained in the two positions for both AoP and HPD, and to predict the change (Δ) in AoP and HPD when transitioning from the dorsal lithotomy to the kneeling squat position, adjusting for the same covariates. Segmented regression analysis was also applied to estimate the intervals for which the increase of the curve can be assumed as constant and the cut‐off thresholds for which there is a change in ΔAoP and ΔHPD^34^.

All statistical tests were two‐sided and the level of significance was set at α = 0.05. The study was reported following the Strengthening the Reporting of Observational Studies in Epidemiology (STROBE) guidelines^35^ and was approved by the ethics committee of the University Hospital of Parma (270/2018/OSS/AOUPR). Statistical analysis was conducted using the statistical software package R version 4.5.2 (R Foundation for Statistical Computing, Vienna, Austria).

## RESULTS

Overall, 78 patients provided informed consent for study enrolment, of whom 55 were included following sequential transperineal assessment of fetal head station, first in the dorsal lithotomy position and then in the kneeling squat position. The maternal demographics and labor outcomes of the included cases are shown in Table 1. Compared using the Wilcoxon signed‐rank test, a wider mean AoP was recorded in the kneeling squat position compared with the dorsal lithotomy position (135.4° ± 15.1° *vs* 125.6° ± 11.9°; *P* < 0.01). This difference remained significant after adjustment for covariates (β, 9.78 (95% CI, 7.03–12.53); *P* < 0.01) (Table 2, Figure S1a). Consistently, the mean HPD was significantly shorter in the kneeling squat position compared with the dorsal lithotomy position (30.3 ± 8.2 *vs* 35.5 ± 8.9 mm; *P* < 0.01). Again, this difference remained significant after adjusting for covariates using a linear mixed‐effects model (β, −5.2 (95% CI, −6.9 to −3.6); *P* < 0.01) (Table 2, Figure S1b). Moreover, a shorter HPD was recorded in fetuses in the occiput anterior position compared with those in the non‐occiput anterior position (β, −12.8, (95% CI, −23.7 to −1.9); *P* = 0.02) after adjusting for the other covariates (Table 2).

**Table 1:** Maternal demographic characteristics and labor outcomes of the included cases (*n* = 55)

Characteristic	Value
Ethnicity	
White	50 (90.9)
African	2 (3.6)
Asian	3 (5.5)
Nulliparous	43 (78.2)
Gestational age (weeks)	39 + 6 ± 1 + 2
BMI at presentation (kg/m^2^)	23.1 ± 4.3
Induction of labor	23 (41.8)
Epidural	52 (94.5)
Mode of delivery	
Spontaneous	43 (78.2)
Instrumental	12 (21.8)
Duration of labor (h)	7.6 ± 3.4
Clinical fetal head station*	
≤ −1	30 (54.5)
0	19 (34.5)
≥ +1	6 (10.9)
Occiput anterior position*	53 (96.4)
Postpartum hemorrhage	11 (20.0)
Episiotomy	8 (14.5)
OASI	2 (3.6)
Birth weight (g)	3290 ± 475
1‐min Apgar score	9 (8–9)
5‐min Apgar score	10 (9–10)
Arterial pH	7.2 ± 0.1
Arterial base excess (mEq/L)	−5.6 ± 2.7
NICU admission	9 (16.4)

Data are given as *n* (%), mean ± SD or median (range).

* Clinical assessment of fetal head station and occiput anterior position was performed with patient in dorsal lithotomy position. BMI, body mass index; NICU, neonatal intensive care unit; OASI, obstetric anal sphincter injury.

**Table 2:** Estimated effect (β) of maternal position and other predictor variables on angle of progression and head‐to‐perineum distance measured via intrapartum transperineal ultrasound

	Angle of progression		Head‐to‐perineum distance	
Predictor	β (95% CI)	*P*	β (95% CI)	*P*
Kneeling squat *vs* dorsal lithotomy maternal position	9.78 (7.03 to 12.53)	< 0.01	−5.2 (−6.9 to −3.6)	< 0.01
Ethnicity*	10.73 (−0.52 to 21.98)	0.06	−3.3 (−10.4 to 3.8)	0.36
Occiput anterior position†	17.21 (−0.16 to 34.57)	0.05	−12.8 (−23.7 to −1.9)	0.02
Parity‡	−0.47 (−8.33 to 7.39)	0.91	4.5 (−0.4 to 9.5)	0.07

Results were obtained from linear mixed‐effects models and adjusted for the other predictor variables in the model.

* White *vs* non‐White ethnicity.

† Occiput anterior *vs* non‐occiput anterior position.

‡ Parous *vs* nulliparous.

**Figure 2:**
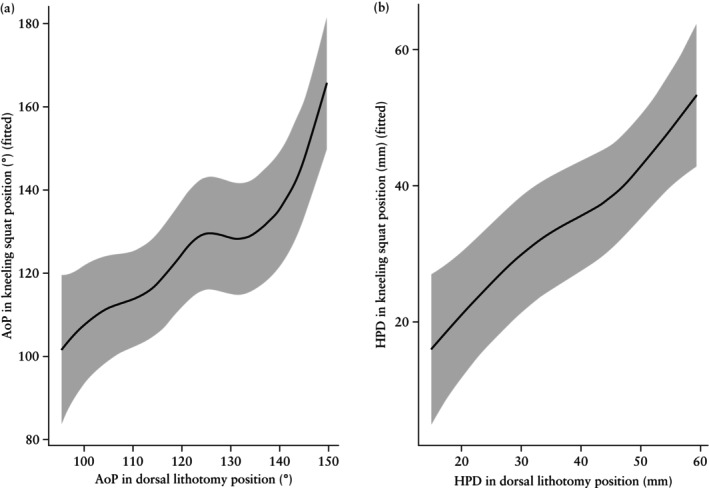
Smooth term graphs showing the relationship between measurements of angle of progression (AoP) (a) and head‐to‐perineum distance (HPD) (b) in kneeling squat *vs* dorsal lithotomy position. Curves were obtained using generalized additive models (GAMs), adjusted for ethnicity, parity and fetal occiput position. Shaded area represents 95% CI.

Smooth term graphs, derived using GAMs after adjustment for covariates, show the increasing non‐linear relationship between AoP (Figure 2a) and HPD (Figure 2b) measurements obtained in the dorsal lithotomy and kneeling squat positions. GAMs adjusted for covariates were also used to predict the AoP and HPD in the kneeling squat position based on the measurement in the dorsal lithotomy position (Tables S1 and S2).

Segmented regression analysis was applied to document the changes in the AoP from the dorsal lithotomy to the kneeling squat position and showed an initial slope of 0.95. After the first breakpoint when the AoP was 121.5° in the dorsal lithotomy position, the slope decreased by 0.49, resulting in a new slope of 0.46. When the AoP was beyond 145.2° in the dorsal lithotomy position, the slope increased sharply by 7.46, indicating that for an observed increase in AoP of 1° in the dorsal lithotomy position there is an average increase of 7.92° in the kneeling squat position (Figure S2a). For the HPD, a similar pattern emerged, but with only one breakpoint; the initial slope was 0.63, which increased by 1.53 beyond a HPD breakpoint set at 48.4 mm in the dorsal lithotomy position (Figure S2b).

## DISCUSSION

### Main findings

Within an unselected cohort of women who underwent intrapartum transperineal sonographic assessment of fetal head station, prior to and following maternal mobilization from the dorsal lithotomy position to the kneeling squat position during the second stage of labor, the sonographic indicators of fetal head descent (AoP and HPD) are more favorable in the kneeling squat position compared with the dorsal lithotomy position. The extent of the increase in the sonographic fetal head station in the kneeling squat position may be predicted based on the sonographic fetal head station observed prior to mobilization (i.e. in the dorsal lithotomy position). In addition, based on our data, the sonographic fetal head station following mobilization may be impacted by the fetal occiput position.

### Comparison with the literature

Pelvic size is among the parameters that may impact labor progression^19^, ^28^, ^30^, ^36^, ^37^, ^38^, ^39^. Maternal mobilization may allow a small increase of the pelvic diameters, which can be relevant to labor management^40^, ^41^. The existing evidence on the impact of intrapartum horizontal and vertical positions on the maternal pelvis is based on the use of dynamic external pelvimetry tests, magnetic resonance imaging and modified computational models, all of which have consistently demonstrated more favorable pelvic diameters in vertical maternal positions^42^. More specifically, when in a maternal vertical position, the inclination of the pelvic inlet edge supports fetal head descent and the transverse diameter of the pelvic outlet increases to facilitate birth^42^. A study conducted using magnetic resonance imaging showed that transitioning from the supine dorsal position to the kneeling squat position increases the transverse diameter of the midpelvic region and the pelvic outlet^36^. Additionally, Michel *et al*.^29^ observed in non‐pregnant women that adopting the hands‐and‐knees position significantly increased the anteroposterior pelvic outlet and bispinous diameters compared with the supine position^29^, ^31^. Moreover, in vertical positions, the sacrum is unloaded and, consequently, coccyx mobilization is maximal^43^. Therefore, from a biomechanical perspective, the kneeling squat position can increase the pelvic outlet diameter, enhance sacral flexibility and facilitate gravitational assistance of fetal head descent^43^.

To our knowledge, only one other study has assessed the impact of maternal mobilization on fetal head station sonographically. Cuerva *et al*.^32^ compared intrapartum ultrasound measurements of AoP and HPD, taken during and following uterine contractions, in the maternal lateral position *vs* the semirecumbent position. Consistent with our data, small but significant differences in measurements between the two positions were observed following mobilization, suggesting indicators of fetal head station are more favorable in the lateral position compared with the semirecumbent position. However, the study did not provide evidence to suggest that the sonographic fetal head station following mobilization can be predicted based on the fetal head station observed in the prior semirecumbent position^32^.

With respect to the differences in HPD following mobilization in relation to the position of the fetal occiput, it is known that the fetal head follows a different direction of descent into the birth canal depending on its position^44^; more specifically, at the midcavity level, in the event of occiput anterior position, the fetal head has a horizontal or upward direction, while in the event of occiput posterior position, the head is still in a downward orientation^44^. A previous study from our group suggested that the HPD represents the most reliable parameter measured using transperineal ultrasound for the assessment of fetal head station, given that it is not dependent upon the direction of the head descent^45^. In such context, the results from this study suggest that maternal mobilization may impact on the level of the presenting part differently depending on the position of the fetal occiput.

### Clinical and research implications

Intrapartum ultrasound, specifically transperineal ultrasound, represents the most accurate and reproducible tool for the assessment of fetal head station and position in a clinical setting^46^, ^47^. To our knowledge, limited data exist on the impact of maternal mobilization during labor on fetal head station assessed using transperineal s onography. The present study shows that maternal mobilization from the dorsal lithotomy position to the kneeling squat position is associated with more favorable indicators of fetal head station, particularly in the context of occiput anterior position. This may be explained by the fact that maternal mobilization to a vertical position is associated with increased size of the obstetric conjugate and other diameters of the birth canal. Another explanation is that maternal mobilization affects the relative position of the presenting part with respect to the pubic symphysis, which is imaged to obtain the measurement of the AoP. Therefore, the more favorable sonographic fetal head station observed in kneeling squat position may result either from a true descent facilitated by gravity and increased diameters of the birth canal or, as an alternative explanation, a different anatomical relation between the presenting part and the sonographic landmarks of the birth canal, as evaluated using transperineal ultrasound.

Based on our data, another hypothesis is that the position of the fetal occiput may determine whether mobilization from the dorsal lithotomy position to the kneeling squat position supports or precludes fetal head descent. The unexpectedly small number of fetuses in a non‐occiput anterior position in this study is to be considered a limitation; therefore, the actual role of maternal mobilization in relation to the position of the fetal occiput should be reinvestigated in larger prospective studies.

The present study does not provide evidence regarding the impact of maternal mobilization on clinically meaningful outcomes on which the recommendations regarding maternal position in labor should be based. However, the findings suggest that the kneeling squat position may promote the descent of the fetal head into the maternal pelvis, particularly in the event of occiput anterior position, which encourages further investigation into the role of maternal mobilization in labor management through prospective observational studies or RCTs.

In addition, the latent phase of the second stage of labor may represent a valuable opportunity to mobilize the patient and encourage positions that may enhance pelvic mobility and size and facilitate fetal head descent during maternal pushing efforts. More favorable indicators of fetal head descent in the kneeling squat position suggest that maternal mobilization during the latent phase of the second stage of labor may avoid early and non‐effective pushing efforts and allow passive descent of the fetal head. This could also be beneficial for the laboring woman in terms of recovery and energy preservation for the active phase of the second stage of labor. It is important to note that the data from this study are unlikely to be of relevance in cases in which an obstetric intervention is indicated in the second stage of labor, as instrumental delivery is routinely performed with patients in the dorsal lithotomy position.

### Strengths and limitations

The main strength of this study lies in its original and prospective design involving sequential measurements of two sonographic indicators of fetal head station in two different maternal positions. Moreover, this study was conducted by a team with expertise in intrapartum ultrasound and labor management.

With respect to the limitations, the small number of cases and the inclusion of a vast majority of cases with the fetus in occiput anterior position is to be acknowledged, which may be related to the fact that only cases with normal labor progression were included. Furthermore, the non‐randomized order of the sonographic assessment determines a risk of systematic order‐related bias. We also acknowledge that, irrespective of adequate training in intrapartum sonography, performing transperineal ultrasound with the patient in kneeling squat position may be challenging. Furthermore, to our knowledge, no previous data exist on the reproducibility of AoP and HPD measurements obtained in vertical maternal positions. Finally, the single‐center design may reduce the generalizability of the study findings, and the lack of investigation of clinically meaningful outcomes precludes determination of their clinical relevance.

### Conclusion

This study suggests that, during the latent phase of the second stage of labor, maternal mobilization into upright positions, such as the kneeling squat position, is associated with improved sonographic indicators of fetal head station. However, this study only supports such findings in the event of fetal occiput anterior position. Sonographic studies with larger cohorts are warranted to evaluate the clinical relevance of these findings, as well as the role of maternal mobilization in the second stage of labor in relation to the position of the fetal occiput.

## Supporting information


**Figure S1** Comparison between mean ± SD angle of progression (AoP) (a) and head‐to‐perineum distance (HPD) (b) measurements in dorsal lithotomy and kneeling squat positions. Results were obtained using linear mixed‐effects models and were adjusted for ethnicity, parity and occiput position. *P < 0.05.

**Figure S2** Segmented regression analysis showing changes in measurements of angle of progression (AoP) (a) and head‐to‐perineum distance (HPD) (b) when transitioning from dorsal lithotomy position to kneeling squat position (fitted values), adjusted for ethnicity, parity and occiput position. Shaded area represents 95% CI.

**Table S1** Prediction of angle of progression (AoP) in kneeling squat position based on initial measurement recorded in dorsal lithotomy position. Values were obtained using generalized additive models and adjusted for ethnicity, parity and occiput position.

**Table S2** Prediction of head‐to‐perineum distance (HPD) in kneeling squat position based on initial measurement recorded in dorsal lithotomy position. Values were obtained using generalized additive models and adjusted for ethnicity, parity and occiput position.

## Data Availability

The data that support the findings of this study are available on request from the corresponding author. The data are not publicly available due to privacy or ethical restrictions.
