# Supplementary figures and images for: Intrapartum sonographic evaluation of fetal head descent in relation to maternal position: comparison between dorsal lithotomy and kneeling squat positions
Source: Ultrasound Obstet Gynecol. 2026 Apr 15;67(5):665–71. doi: 10.1002/uog.70230 (PMC13136051; doi:10.1002/uog.70230)

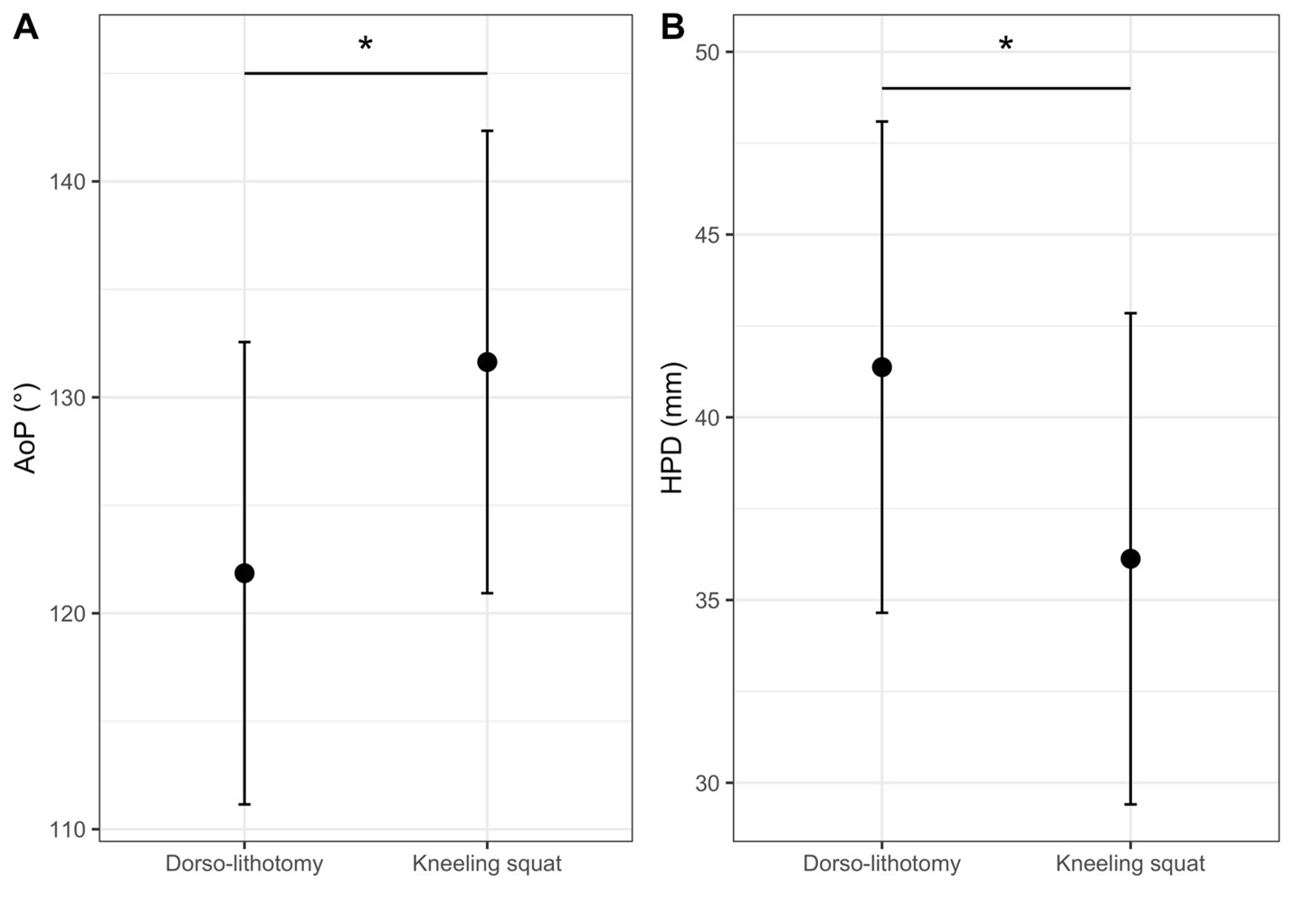

Supplement: Supplementary file 1 — Figure S1 Comparison between mean ± SD angle of progression (AoP) (a) and head‐to‐perineum distance (HPD) (b) measurements in dorsal lithotomy and kneeling squat positions. Results were obtained using linear mixed‐effects models and were adjusted for ethnicity, parity and occiput position. *P < 0.05. [file UOG-67-665-s002.jpg]

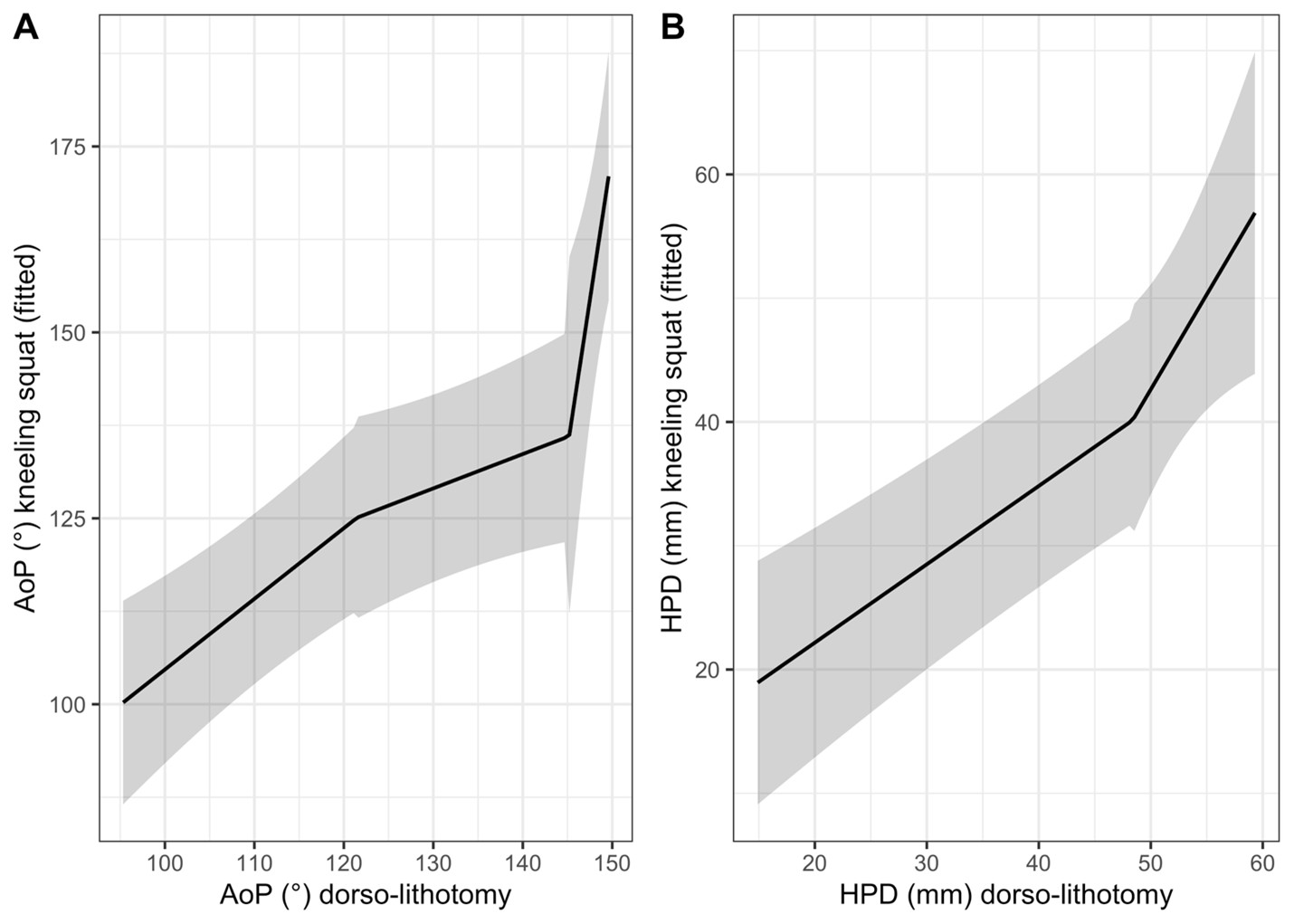

Supplement: Supplementary file 2 — Figure S2 Segmented regression analysis showing changes in measurements of angle of progression (AoP) (a) and head‐to‐perineum distance (HPD) (b) when transitioning from dorsal lithotomy position to kneeling squat position (fitted values), adjusted for ethnicity, parity and occiput position. Shaded area represents 95% CI. [file UOG-67-665-s004.jpg]
