# Supplementary material for: Intrapartum sonographic evaluation of fetal head descent in relation to maternal position: comparison between dorsal lithotomy and kneeling squat positions
Source: Ultrasound Obstet Gynecol. 2026 Apr 15;67(5):665–71. doi: 10.1002/uog.70230 (PMC13136051; doi:10.1002/uog.70230)
Supplement: Supplementary file 4 — Table S2 Prediction of head‐to‐perineum distance (HPD) in kneeling squat position based on initial measurement recorded in dorsal lithotomy position. Values were obtained using generalized additive models and adjusted for ethnicity, parity and occiput position. [file UOG-67-665-s001.docx]

**Table S2** Prediction of head-to-perineum distance (HPD) in kneeling squat position based on initial measurement recorded in dorsal lithotomy position. Values were obtained using generalized additive models and adjusted for ethnicity, parity and occiput position.

| **HPD in dorsal lithotomy (mm)** | **HPD in kneeling squat (mm)** |
| --- | --- |
| 14.9 | 13.7 |
| 19.8 | 18.7 |
| 24.8 | 23.3 |
| 29.7 | 27.5 |
| 34.6 | 30.7 |
| 39.6 | 33.2 |
| 44.5 | 35.8 |
| 49.4 | 40.1 |
| 54.4 | 45.4 |
| 59.3 | 51.1 |
